# Partial Inhibition of Estrogen-Induced Mammary Carcinogenesis in Rats by Tamoxifen: Balance between Oxidant Stress and Estrogen Responsiveness

**DOI:** 10.1371/journal.pone.0025125

**Published:** 2011-09-26

**Authors:** Bhupendra Singh, Nimee K. Bhat, Hari K. Bhat

**Affiliations:** Division of Pharmacology and Toxicology, School of Pharmacy, University of Missouri-Kansas City, Kansas City, Missouri, United States of America; University of Alabama at Birmingham, United States of America

## Abstract

Epidemiological and experimental evidences strongly support the role of estrogens in breast tumor development. Both estrogen receptor (ER)-dependent and ER-independent mechanisms are implicated in estrogen-induced breast carcinogenesis. Tamoxifen, a selective estrogen receptor modulator is widely used as chemoprotectant in human breast cancer. It binds to ERs and interferes with normal binding of estrogen to ERs. In the present study, we examined the effect of long-term tamoxifen treatment in the prevention of estrogen-induced breast cancer. Female ACI rats were treated with 17β-estradiol (E2), tamoxifen or with a combination of E2 and tamoxifen for eight months. Tissue levels of oxidative stress markers 8-iso-Prostane F_2α_ (8-isoPGF_2α_), superoxide dismutase (SOD), glutathione peroxidase (GPx), catalase, and oxidative DNA damage marker 8-hydroxydeoxyguanosine (8-OHdG) were quantified in the mammary tissues of all the treatment groups and compared with age-matched controls. Levels of tamoxifen metabolizing enzymes cytochrome P450s as well as estrogen responsive genes were also quantified. At necropsy, breast tumors were detected in 44% of rats co-treated with tamoxifen+E2. No tumors were detected in the sham or tamoxifen only treatment groups whereas in the E2 only treatment group, the tumor incidence was 82%. Co-treatment with tamoxifen decreased GPx and catalase levels; did not completely inhibit E2-mediated oxidative DNA damage and estrogen-responsive genes *monoamine oxygenase B1* (*MaoB1*) and *cell death inducing DFF45 like effector C* (*Cidec*) but differentially affected the levels of tamoxifen metabolizing enzymes. In summary, our studies suggest that although tamoxifen treatment inhibits estrogen-induced breast tumor development and increases the latency of tumor development, it does not completely abrogate breast tumor development in a rat model of estrogen-induced breast cancer. The inability of tamoxifen to completely inhibit E2-induced breast carcinogenesis may be because of increased estrogen-mediated oxidant burden.

## Introduction

Sex hormones have been implicated in the development of breast cancer [1], [2], [3]. However, the exact mechanisms underlying the initiation and progression of estrogen-related cancers remain elusive. Breast tumor induction is suggested to depend on both the estrogen receptor (ER)-dependent pathway of estrogen-induced cell growth and ER-independent pathway that involves estrogen metabolism and oxidative stress resulting from the redox cycling of estrogen metabolites [1], [4], [5], [6]. Published literature and our recent studies strongly support the role of estrogen metabolism-mediated oxidative stress in estrogen-induced breast carcinogenesis [1], [2], [7], [8], [9].

Tamoxifen (Tam) is a cancer chemotherapeutic agent of a drug family known as ER modulators and is widely used as an anticancer and chemopreventive drug for breast cancer [10], [11]. The molecular mechanism(s) underlying the tissue-specificity of Tam action is not clear. The antagonist effects of Tam in breast tissue are thought to result from its ability of competing with estradiol to bind to the ligand-binding domain of the ER and by inducing conformational changes that block the interaction of ER with coactivator proteins [12]. Tamoxifen is known to induce a significant improvement in the overall survival rate from breast cancer [13]. However, about one-third of ER and progesterone receptor (PR) positive breast tumors treated with Tam do not respond to initial treatment, and the remaining 70% are still at risk to relapse in the future [14]. An increased incidence of endometrial cancer in breast cancer patients and induction or promotion of tumorigenesis in human and rat liver treated with Tam has also been reported [15], [16]. The development of endocrine resistance is a pervasive clinical problem [17], [18]. A number of mechanisms have been proposed to control antiestrogen resistance in ER-positive breast cancer, but many details of these mechanisms continue to be unclear [19]. Moreover, Tam itself has been shown to possess pro-carcinogenic properties and strong genotoxic activity both *in vivo* and *in vitro* [20]. Tamoxifen appears to require metabolic activation to exert tumorigenic and genotoxic activity [21]. A number of earlier studies have shown that Tam is converted to the genotoxic epoxide, as well as 4- and 2-hydroxy metabolites *via* enzymatic activation either by liver cytochrome P450 monoxidase [22] or by different peroxidases [23], [24]. These metabolites can bind to biologically active molecules like DNA, lipids and proteins, and are capable of inducing irreversible damage to these molecules [25]. Overall, the mechanisms of action of Tam depend upon several interlinked factors and are not well understood. It has previously been reported that Tam treatment for 6 months abrogates E2-induced breast cancer [26]. In the present study, we report that long-term (8 months) Tam treatment does not completely prevent E2-induced breast cancer in ACI rat breast cancer model, an accepted animal model of hormonal breast cancer. The lack of complete inhibition of E2-induced breast cancer by Tam may be because of the inability of Tam to prevent E2-mediated oxidant stress as is suggested by the current studies.

## Results

### Tamoxifen decreases breast tumor incidence and increases the latency of estrogen-induced mammary tumor development

**Figure 1:**
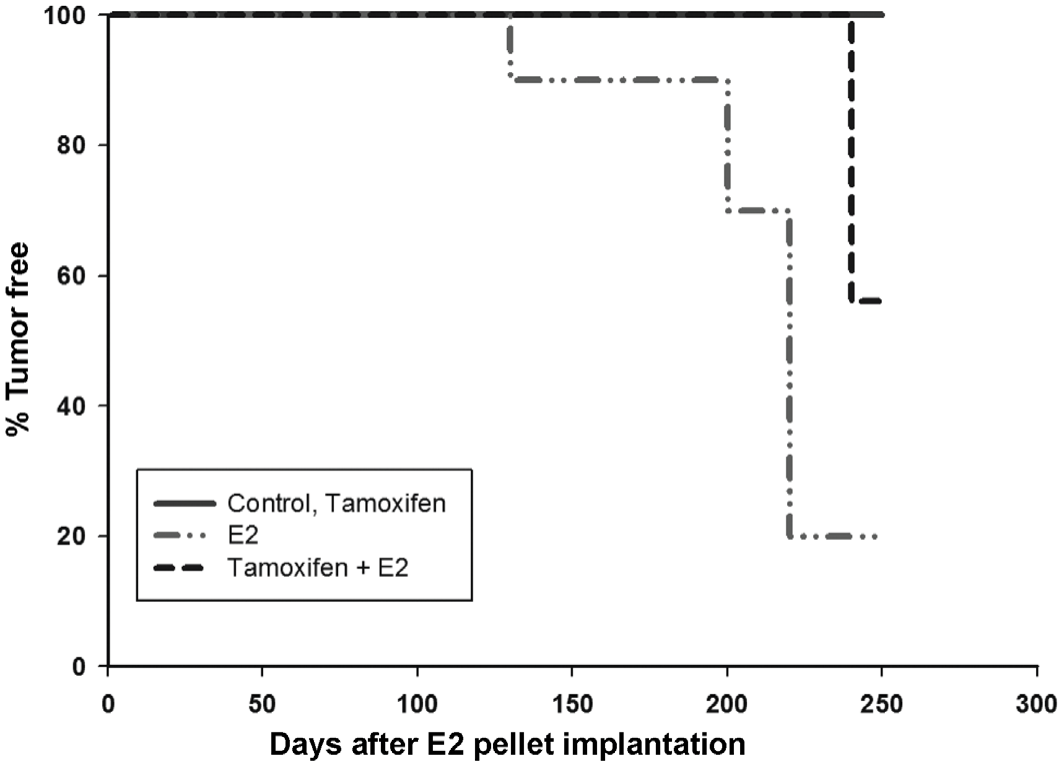
Tamoxifen exposure increases the latency of E2-induced breast tumors. Female ACI rats were treated with E2, Tam or Tam + E2 as described in the Materials and Methods section. Kaplan-Meier survival curves for tumor occurrence were plotted for each treatment group, and the log rank test was used to detect differences in tumor latency curves between groups. Average tumor latency was significantly longer for animals in the Tam + E2 group versus those in the E2 group. Animals in the control or Tam groups did not develop any tumors and are represented by the same line on the graph.

**Figure 2:**
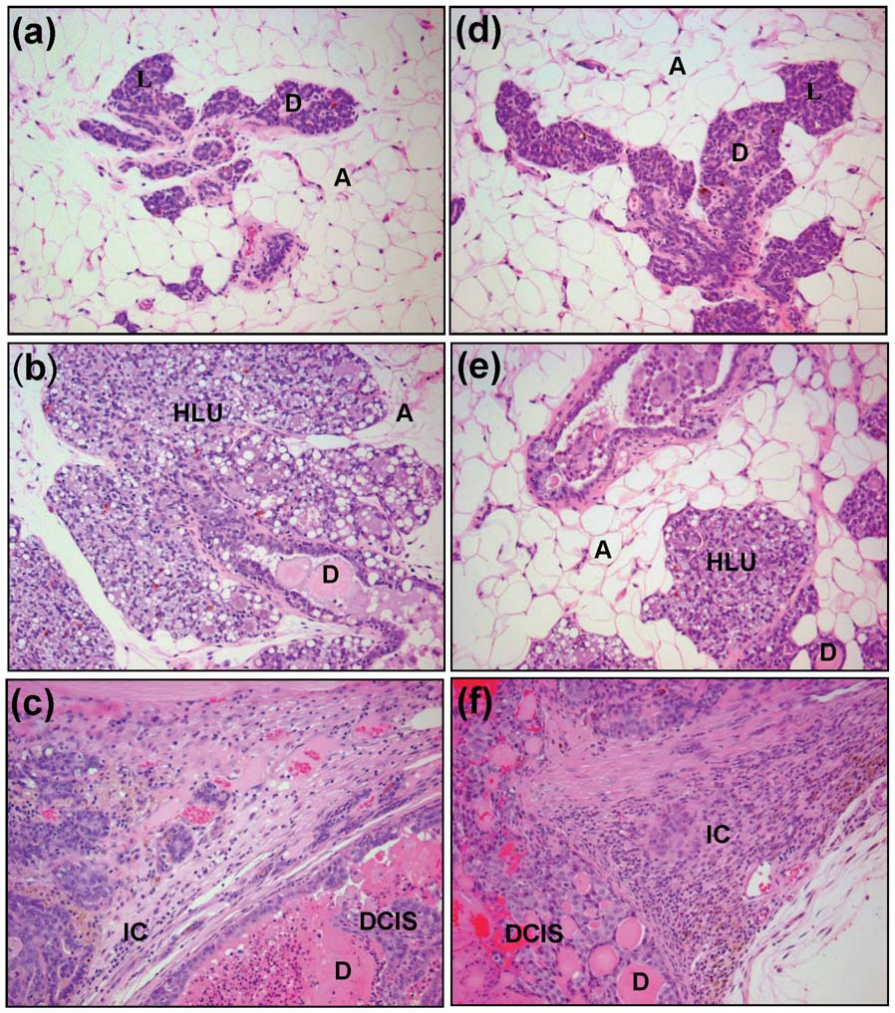
Histopathology of mammary tumor and mammary tissues. Female ACI rats were treated with E2, Tam or Tam + E2 for 240 days as described in the Materials and Methods section. a) The mammary tissue of a representative control ACI rat shows normal lobular architecture (L) with branched ducts (D) and normal distribution of fat tissue/adipocytes (A); b) E2-treated mammary tissue shows increased proliferation with dilated ducts containing inspissated secretions (D) and increased proliferation and expansion of terminal lobular units (HLU) accompanied by compression of and expansion into the surrounding fat tissue/adipocytes (A); c) Mammary tissue from a rat treated with E2 shows ductal carcinoma in situ (DCIS) containing inspissated secretions in ducts (D) and micro-invasive cancer (IC); d) The mammary gland of Tam only treated rat shows normal lobular architecture (L) with branched ducts (D) and normal distribution of fat tissue (A); e) Mammary tissue from Tam + E2-exposed rat displays increased proliferation compared to control mammary tissue but less than E2-treated mammary tissue. It also shows dilated ducts containing inspissated secretions (D) and increased proliferation and expansion of terminal lobular units (HLU) accompanied by compression of and expansion into the surrounding fat tissue (A); f) Mammary tissue from an animal treated with Tam + E2 shows ductal carcinoma in situ (DCIS) containing inspissated secretions in ducts (D) and micro-invasive cancer (IC). 100× magnification.

Female ACI rats were treated with E2, Tam or Tam + E2 for 8 months. Control animals were sham-operated and received cholesterol pellet only which was used as a binder for the preparation of E2 pellets. Mammary tumor incidence in rats co-treated with E2 and Tam at necropsy was 44% (Table 1). No palpable tumors were observed in this group till the end of the treatment time. In contrast, in E2-treated group, the first palpable breast tumors appeared after 128 days of treatment and mammary tumor incidence was 82% after 8 months of E2 exposure [7]. Kaplan-Meier survival curve analysis suggests that average tumor latency was significantly longer for animals in the Tam + E2 group versus those in E2 group (Figure 1). Co-treatment with Tam and E2 was associated with a decrease in tumor multiplicity compared to E2 group (Table 1). In the Tam + E2 group, 1.4±0.3 tumor nodules per tumor-bearing animal were observed while in the E2 treatment group, an average of 3.1±0.7 tumor nodules were present in tumor-bearing rats (Table 1). No tumors were observed in sham-operated controls or in Tam-only treated groups. No morphologic changes were detected in the mammary tissue of Tam-treated ACI rats relative to those of sham controls (Figure 2). Mammary tissues from all the animals in the Tam-treated group displayed normal lobular architecture, consisting of ducts surrounded by small lobules and were similar to that of controls and did not exhibit increased proliferation (Figure 2). Analysis of mammary tissue from all the rats in the Tam + E2 experimental group revealed less lobular and intraductal proliferation compared to rats treated with E2 (Figure 2). However, both ductal carcinoma in situ (DCIS) and micro-invasive cancers were present in the mammary tissue of animals from E2 and Tam + E2 group (Figure 2). The mammary tumors from Tam + E2 group have characteristics similar to E2-induced breast tumors [26], [27], [28]. No significant morphological differences were observed between ovary, kidney, uterus, lung or liver tissues from rats in the Tam and Tam + E2 groups (data not shown).

**Table 1:** Effects of E2 and Tam treatments on mammary tumor development in female ACI rats.

Treatment Groups	n	Tumor Incidence (%)	Tumor Multiplicity	Appearance of First Tumor (day)
Control	10	0	0	NA
E2	11	82*	3.1±0.7*	128*
Tam	18	0	0	NA
Tam + E2	18	44^#^	1.4±0.3^#^	At necropsy

Column 1 lists different treatments each group of animals received. The number of animals per group (*n*) is listed in column 2. Percent tumor incidence after 240 days of treatment period is listed in column 3, and the average number of tumors per tumor-bearing animal (tumor multiplicity) is listed in column 4. Column 5 lists the day on which the first tumor appeared in each group (appearance of first tumor). ‘*’ indicates significant difference (*p*<0.05) compared to control group. ‘#’ indicates significant difference (*p*<0.05) between Tam + E2 and control or Tam-treated group.

### Tamoxifen decreases estrogen and progesterone receptor expression, and E2-induced mammary proliferation

**Figure 3:**
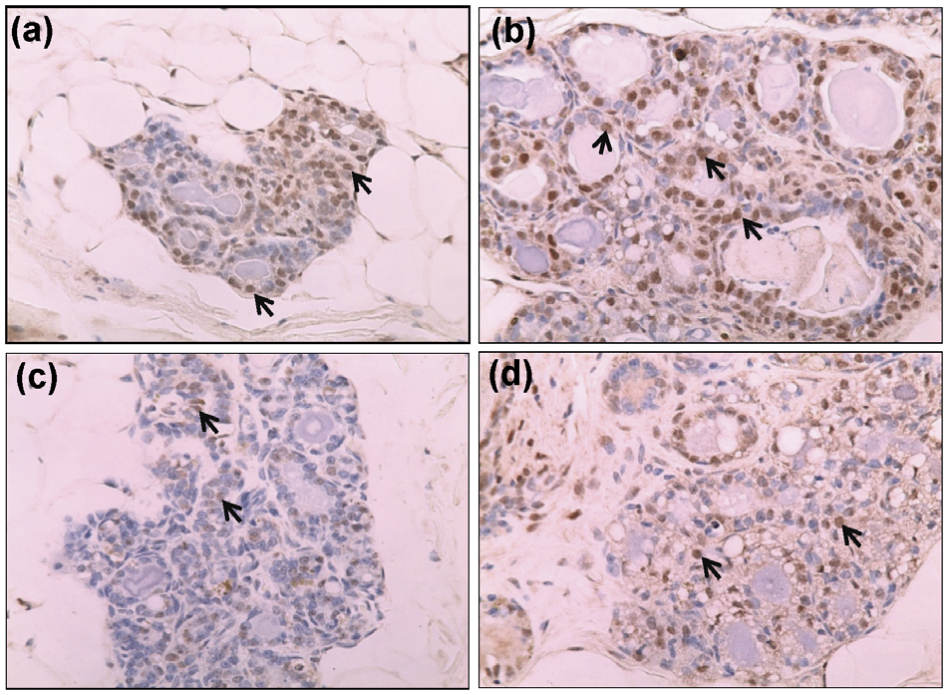
Expression of ER-α in mammary tissues. Female ACI rats were treated with E2, Tam or Tam + E2 for 240 days as described in Materials and Methods section. At the end of the experiment, mammary tissues were collected and used for immunohistochemistry as described in Materials and Methods section. Formalin-fixed/paraffin-embedded mammary sections were immunostained with ER-α antibody. Nuclear expression of ER-α is shown in representative sections (arrow). (a) The mammary tissue of a representative control ACI rat; (b) E2-treated mammary tissue; (c) Tam-treated mammary tissue; and (d) Tam + E2-treated mammary tissue. 100× magnification.

**Figure 4:**
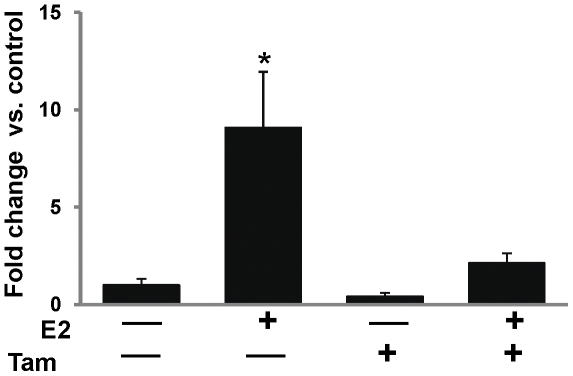
Expression of *PR* mRNA in mammary tissues. Female ACI rats were treated with E2, Tam or Tam + E2 for 240 days as described in Materials and Methods section. At the end of the experiment, mammary tissues were collected, total RNA isolated and used for real-time PCR as described in Materials and Methods section. *PR* mRNA expression was significantly increased in E2-treated mammary tissues and it remained unchanged in Tam- and Tam + E2-treated mammary tissues. mRNA expression data are presented as fold change versus control mammary tissue. These data are reported as an average of values obtained for at least 5 different animals ± SEM. ‘*’ indicates a p value<0.05 compared to controls.

**Figure 5:**
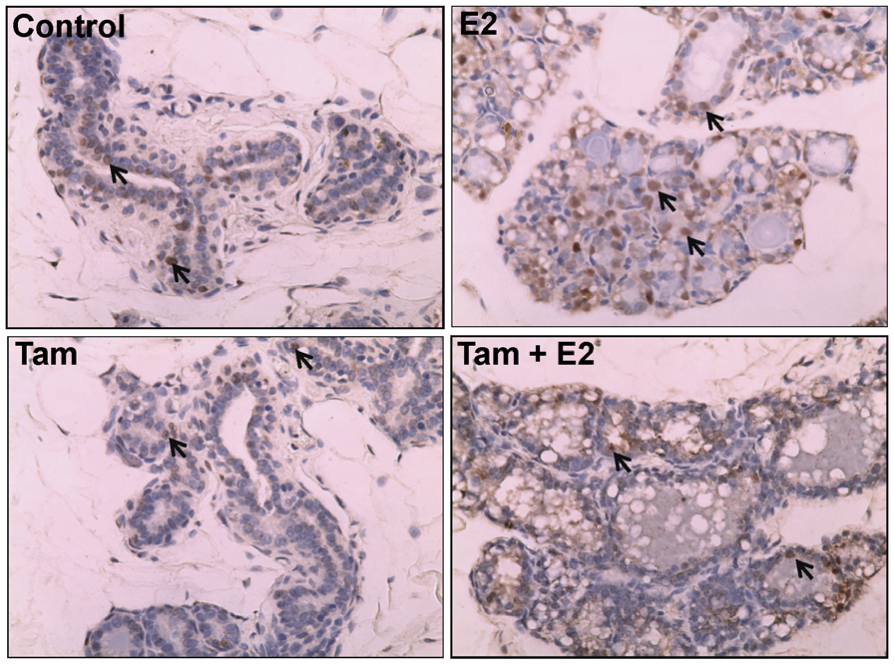
Immunohistochemical detection of proliferation marker PCNA in mammary tissues. Female ACI rats were treated with E2, Tam or Tam + E2 for 240 days as described in Materials and Methods section. At the end of the experiment, mammary tissues were collected in 10% buffered-formalin. Formalin-fixed/paraffin-embedded mammary tissue sections were immunostained with PCNA antibody. Nuclear expression of PCNA is shown in representative sections (arrows) at 100× magnification.

Estrogen receptor-α and *PR* expression was analyzed by immunohistochemical and real-time PCR, respectively. Increased ER-α protein expression was observed in E2-treated mammary tissue than the control mammary tissue by immunohistochemical analysis (Figure 3). In contrast, ER-α protein expression was lower in Tam and Tam + E2-treated mammary tissues compared to control mammary tissues (Figure 3). *Progesterone receptor* mRNA expression was 9-fold upregulated in E2-treated mammary compared to the control mammary (Figure 4). Fold changes in *PR* mRNA expression were not significantly different in Tam and Tam + E2-treated mammary tissues compared to control mammary tissues (Figure 4). Immunohistochemical analysis for proliferating cell nuclear antigen (PCNA) as a marker for proliferating cells (26) showed no significant change in PCNA protein levels between control and Tam-exposed mammary tissues (Figure 5). PCNA expression in Tam + E2-treated mammary tissue was more than the Tam alone group but lower than E2-exposed mammary tissues (Figure 5).

### Tamoxifen does not prevent E2-induced oxidative stress

**Figure 6:**
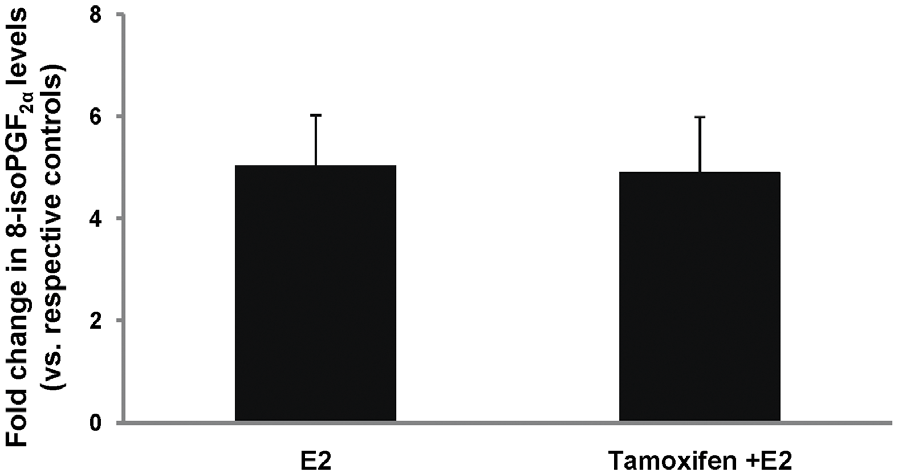
8-iso-prostane F_2α_ (8-isoPGF_2α_) formation in rat mammary tissues. Female ACI rats were treated with E2, Tam or Tam + E2 for 240 days as described in Materials and Methods section. 8-isoPGF_2α_ levels were measured in mammary tissues from animals in each of these groups. Fold changes were calculated for E2-treated animals relative to age-matched cholesterol-treated controls. Fold changes were calculated for Tam + E2-treated animals relative to age-matched Tam-treated controls. The data are reported as an average of fold change values obtained for at least 8 different animals ± SEM.

Oxidative stress in the mammary tissue of ACI rats was evaluated by quantification of 8-isoPGF_2α_ as well as by analysis of antioxidant enzymes SOD, catalase (CAT) and GPx. 8-iso-Prostane F_2α_ levels were significantly greater (∼5-fold) in the Tam + E2 group compared to the Tam control group (Figure 6). In rats treated with E2 only, a similar 5-fold increase in 8-isoPGF_2α_ levels was observed relative to age-matched cholesterol-treated controls. The fold increase in 8-isoPGF_2α_ levels was same in both Tam + E2- and E2-only treated groups (Figure 6). No significant differences in 8-isoPGF_2α_ levels in liver were detected between control animals and those treated with E2 or Tam + E2 (data not shown).

**Figure 7:**
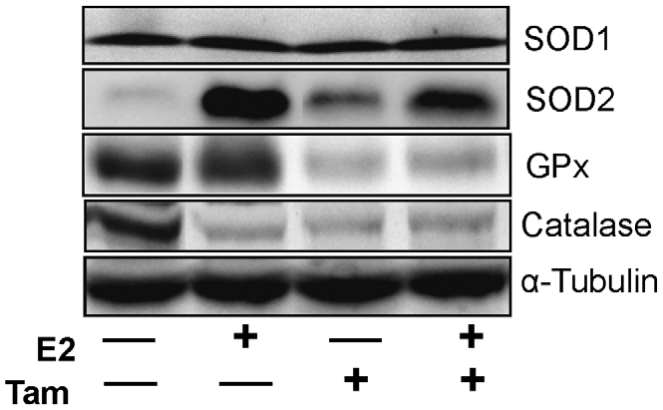
Expression of antioxidant enzymes in mammary tissues. Female ACI rats were treated with E2, Tam or Tam + E2 for 240 days as described in Materials and Methods section. At the end of the experiment, mammary tissues were collected, homogenized and used for western blot analysis. In E2-treated mammary tissue, protein level of CAT is decreased while SOD2 is increased compared to age-matched control. In mammary tissues of rats treated with Tam alone or co-treated with E2, SOD2 is increased, while GPx and CAT are decreased. SOD1 protein levels remain unchanged in all the treatment groups.

Protein expression levels of SODs (SOD1 and SOD2), CAT and GPx in the mammary tissues were evaluated by western blot analysis. There were no significant differences observed in protein expressions levels of SOD1 in Tam-, Tam + E2- and E2-treated mammary tissues compared to control mammary tissues (Figure 7). However, SOD2 protein expression was significantly increased in Tam-, Tam + E2- and E2-treated mammary tissues compared to control mammary tissues (Figure 7). Highest expression of SOD2 was observed in E2-treated mammary tissues (Figure 7). There was no difference in the expression pattern of GPx in control and E2-treated mammary but it was significantly decreased in Tam- and Tam + E2-treated mammary tissues in comparison to control mammary tissues (Figure 7). Catalase expression was significantly decreased in the mammary tissues of all the groups (E2, Tam, Tam + E2) compared to control mammary tissues (Figure 7).

The activities of antioxidant enzymes SOD1, SOD2, CAT and GPx were also quantified in the mammary tissue of rats treated with E2 or Tam + E2 as well as in their respective control groups. Approximately, 3- and 14-fold significant upregulation in SOD1 and SOD2 enzyme activities, respectively were detected in E2-treated mammary tissues compared to control group (Table 2). Tam- and Tam + E2-treated mammary tissues did not show significant change in SOD1 and SOD2 activities compared to controls (Table 2). However, GPx enzyme activities were significantly downregulated in Tam- and Tam + E2-treated mammary tissues (∼2.3 and ∼1.7 fold, respectively) relative to controls (Table 2). No alterations in CAT activity were detected in mammary tissues from any experimental group (Table 2). There was no significant difference in total SOD, CAT and GPx activity in liver tissues between E2 and their control group as well as between Tam + E2 and Tam group (data not shown).

**Table 2:** Activities of antioxidant enzymes in female ACI rats after 240 days of treatment.

Tissue	SOD1 (U/mg protein)	SOD2 (U/mg protein)	GPx (nmol/min/mg protein)	CAT (nmol/min/mg protein)
Control mammary	540±24	40±14	92±9	88±9
E2-treated mammary	1606±280*	568±105*	128±22	104±12
Tam-treated mammary	983±119	35±12	39±10*	79±10
Tam + E2-treated mammary	1034±141	70±27	53±7*	92±14

Female ACI rats were treated with E2, Tam or Tam + E2 as described in the Materials and Methods section. The activities of the antioxidant enzymes SOD1, SOD2, GPx and CAT were measured in mammary tissues from control, E2-, Tam- and Tam + E2-treated rats after 240 days of respective treatment. The data are reported as an average of values obtained for at least 8 different animals ± SEM. ‘*’ indicates a p value<0.05 compared to control mammary tissue.

### Tamoxifen differentially affects Tam-metabolizing enzymes in ACI rat mammary tissues

**Figure 8:**
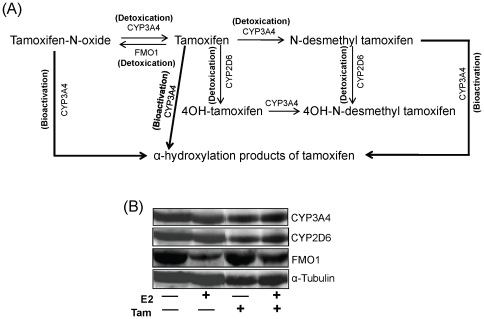
Tamoxifen metabolizing enzymes in mammary tissues. (A) Primary enzymes involved in metabolism of Tam in mammary tissues. Regular arrows indicate the detoxication steps in the pathway, while bold arrows indicate CYP3A4-mediated bioactivation processes. (B) Female ACI rats were treated with E2, Tam or Tam + E2 for 240 days as described in Materials and Methods section. At the end of the experiment, mammary tissues were collected, homogenized and used for western blot analysis. CYP3A4 and CYP2D6 protein levels remain unchanged in all the treatment groups but the FMO1 protein expression is decreased in E2- and Tam + E2-treated mammary tissues.

Cytochrome P450 (CYP) 3A4, CYP2D6 and flavin containing monooxygenase 1 (FMO1) are major Tam-metabolizing enzymes in the body (Figure 8A). Western blotting techniques were employed to evaluate protein levels of CYP3A4, CYP2D6 and FMO1 in mammary tissues of E2-, Tam- and Tam + E2-treated groups and compared to control group (Figure 8B). No significant changes in CYP3A4 and CYP2D6 protein levels were detected in mammary tissues of rats from all the experimental groups relative to controls (Figure 8B). However, western blot analysis of mammary tissues of rats from E2, and Tam + E2 groups showed a marked decrease in FMO1 protein expression levels relative to mammary tissues from control animals (Figure 8B). There was no significant change observed in FMO1 protein expression levels between age-matched control and Tam-treated mammary tissues (Figure 8B).

### Tamoxifen does not prevent inhibition of estrogen responsive genes *MaoB1* and *Cidec*

**Figure 9:**
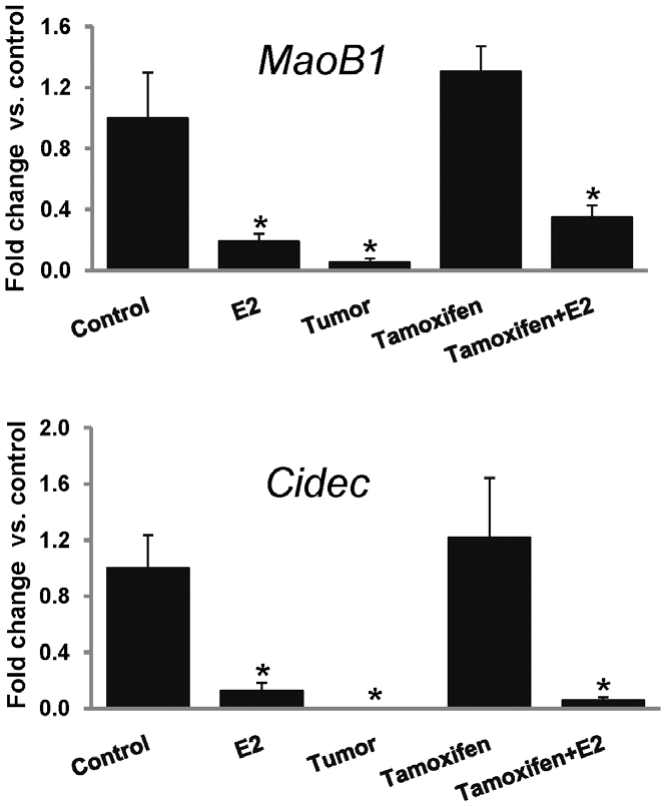
Expression of estrogen regulated genes *MaoB1* and *Cidec* in mammary tissues. Female ACI rats were treated with E2, Tam or Tam + E2 for 240 days as described in Materials and Methods section. mRNA expression levels of *MaoB1* and *Cidec* gene in E2-, Tam- and Tam + E2-treated mammary tissues using quantitative real-time PCR are presented as fold change versus control mammary tissues. Expression of each gene was normalized using cyclophilin as internal control. These data are reported as an average of values obtained for at least 5 different animals ± SEM. ‘*’ indicates a p value<0.05 compared to controls.

Tamoxifen prevented expression of classical E2 responsive genes like *ER-α* and *PR* but did not prevent E2-medaited inhibition of novel E2 responsive genes *MaoB1* and *Cidec* (Figure 9). Both *MaoB1* and *Cidec* mRNA expression levels were significantly downregulated in E2-induced mammary tumors, E2-, and Tam + E2-treated mammary tissues compared to control mammary tissues (Figure 9). However, expression of these genes was unchanged in Tam-treated mammary tissues in comparison to control mammary tissues (Figure 9).

### Tamoxifen does not completely prevent estrogen-induced oxidative DNA damage

**Figure 10:**
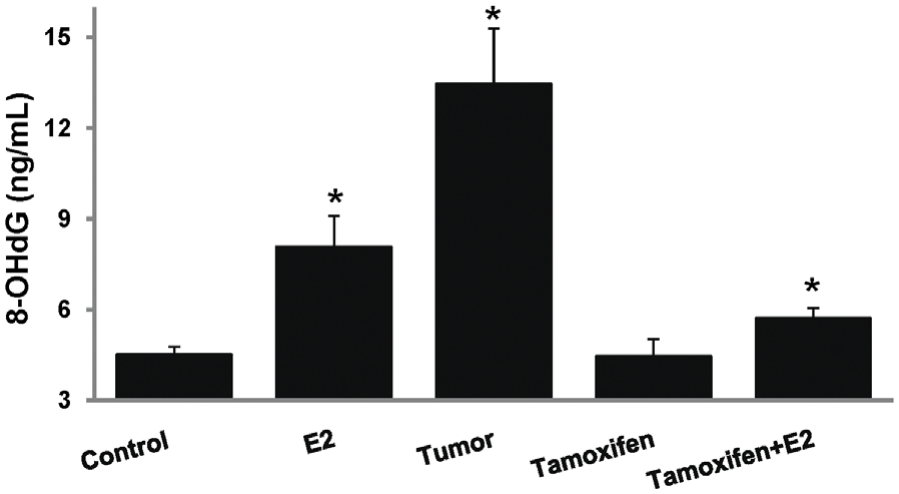
8-hydroxydeoxyguanosine (8-OHdG) levels in mammary tissues and mammary tumors. Female ACI rats were treated with E2, Tam and Tam + E2 for 240 days as described in Materials and Methods section. 8-OHdG levels were measured in mammary tumors and mammary tissues from animals in each of these groups. 8-OHdG levels are significantly increased in E2-treated mammary tissues, mammary tumor tissues and Tam + E2-treated mammary tissues. 8-OHdG levels remain unchanged in Tam-treated mammary tissues compared to control. These data are reported as an average of values obtained for at least 8 different animals ± SEM. ‘*’ indicates a p value<0.05 compared to controls.

Formation of 8-OHdG is a marker of oxidative DNA damage. An ELISA method was used to detect 8-OHdG as a marker of oxidative DNA damage in mammary tissues and mammary tumors. Approximately 1.8- and 3-fold increase (p<0.05) in 8-OHdG levels was observed in E2-treated mammary and in mammary tumors, respectively compared to control mammary (Figure 10). No difference in 8-OHdG levels between control and Tam-treated mammary tissues were observed, suggesting that Tam alone treatment did not induce oxidative DNA damage (Figure 10). However, 8-OHdG levels were significantly increased in Tam + E2-treated mammary tissues relative to control and Tam-only treated mammary tissues (Figure 10).

## Discussion

In present study, we have demonstrated that Tam treatment did not completely abrogate E2-induced mammary tumors in female ACI rats. Female ACI rat model is an established animal model of hormonal carcinogenesis [7], [8], [9], [26], [29], [30], [31]. It shares many features of human breast cancer such as genomic instability, increased oxidant stress, DNA damage etc. [26], [27], [28], [32], [33], [34]. In an earlier report, Tam has been shown to completely abrogate E2-induced breast cancer in the same ACI rat model [26]. This difference may be because of the increased treatment time in our studies (8 months) compared to the earlier published study (6 months). The difference in the outcome could not possibly be due to the lack of Tam supplementation after 6 months because we recovered remaining part of the Tam tablet after 8 months of exposure. Furthermore, Tam did not inhibit E2-induced oxidant stress. However, Tam co-treatment with E2 increased tumor latency and decreased tumor incidence in ACI rat breast (Figure 1). We also demonstrated that Tam inhibited E2-mediated proliferation of mammary tissue (Figure 2). Moreover, low expression levels of PCNA in Tam-treated mammary tissues further confirm the histopathological observations of inhibition of E2-induced proliferation of breast cancer by Tam (Figure 5).

Tamoxifen is a well known selective estrogen receptor modulator (SERM) that inhibits estrogen signaling by competing with estrogen in binding to ERs [12]. The classical pathway of estrogen signaling works through binding of estrogen to ERs and subsequent regulation of estrogen signaling molecules such as PR, Myc etc. [35], [36]. Estrogen receptor and PR regulate an array of genes involved in cell proliferation and oncogenesis [35]. We observed E2-mediated increase in ER and PR expression and inhibition of these receptors by Tam (Figures 3 and 4). Estrogen also exerts its effect by receptor independent estrogen metabolic pathways [1], [5], [37]. During estrogen metabolism, estrogen is converted to highly toxic metabolite 4-hydroxy estradiol (4-OHE2) via CYP1B1 enzymatic reaction [38]. 4-hydroxy estradiol can directly bind to DNA and form DNA adducts [39], [40]. Furthermore, 4-OHE2 metabolizes to semiquinone and quinone [41]. During this conversion of semiquinone to quinone, reactive oxygen species (ROS) are produced that can damage DNA, protein and lipids and ultimately leads to oncogenesis [37], [40], [41]. We have demonstrated that although Tam was able to inhibit ER-mediated processes, it was unable to reduce oxidative stress produced by estrogen (Figure 6). Tamoxifen itself has been shown to induce ROS, and if this is the case one would expect a cumulative effect of E2 and Tam co-treatment on ROS generation [24], [42]. Antioxidants SOD, GPx and CAT expression levels in mammary tissues of Tam- and Tam + E2-treated groups provide an insight to involvement of Tam in regulation of these antioxidant enzymes (Figure 7). Although, SOD1 protein expression levels did not change significantly between E2, Tam, and Tam + E2 groups, GPx expression was markedly decreased in Tam and Tam + E2 group compared to E2-treated group (Figure 7). SOD catalyzes the dismutation of superoxide to generate hydrogen peroxide, while CAT and GPx are responsible for the decomposition of hydrogen peroxide to oxygen and water. No change in SOD1 and increase in SOD2 expression (thus, overall increased SOD levels) without parallel increases in GPx and CAT activity in Tam- and Tam + E2-treated mammary tissues compared to control mammary tissues may result in elevated hydrogen peroxide production. Without sufficient GPx and CAT enzymes available, it may magnify the burden of oxidative stress in the mammary tissues. It may be that long-term Tam treatment, perhaps itself alters the antioxidant status of the mammary in such a way that helps in generation of oxidative stress.

Estrogen has been shown to induce DNA damage and altered antioxidant status of the cells has also been shown to be responsible for increased DNA damage [43]. To further determine if E2-mediated change in antioxidant status of mammary is also reflected in DNA damage, we analyzed the 8-OHdG levels as a marker of oxidative DNA damage in mammary tumors and mammary tissues. We demonstrated significant increased levels of 8-OHdG in Tam + E2-, E2-treated mammary tissues and in mammary tumors compared to control mammary tissue (Figure 10). We also demonstrated that although Tam co-treatment decreased mammary 8-OHdG levels compared to E2 alone treated mammary tissues, it did not bring them to control levels. They were still significantly higher compared to Tam-treated or sham controls. In an earlier study, Tam + E2 treatment for 7 months has been shown to be involved in reduction of 8-OHdG levels in rat mammary [44].

Evidence presented in this study further demonstrates that risks or benefits of Tam treatment depend on the rate of metabolic activation versus detoxification of Tam in the system (Figure 8). CYP3A4, CYP2D6 and FMO1 are well known enzymes involved in metabolic conversion and bioactivation or detoxication of Tam [45], [46], [47], [48], [49], [50]. CYP3A4 bioactivates Tam to N-desmethyl Tam and further to α-hydroxylation products (Figure 8A) that have DNA damaging capabilities [45], [47]. Conversely, CYP2D6 and FMO1 have been shown to be involved in detoxication of Tam and its metabolites [46], [50]. No change in CYP3A4 and CYP2D6 expression and marked decrease in FMO1 protein expression in Tam + E2-treated mammary tissues indicate a change towards more bioactivation of Tam that may be responsible for generation of DNA damaging metabolites (Figure 8B). Furthermore, downregulation of FMO1 in E2- and Tam + E2-treated mammary and no change in the mammary of Tam alone treated group compared to control mammary, indicates an E2-mediated downregulation of this enzyme (Figure 8B).

About two-thirds of human breast cancers that respond to ER-antagonist treatment initially may become somewhat unresponsive to such treatment eventually [18], [51]. The antagonist effects of Tam in breast tissue are thought to result from its ability of competing with estradiol to bind to the ERs [12], [52]. Inhibition of expression of ER and PR in mammary tissues after 8 months of Tam treatment demonstrates continued anti-estrogenic effects of Tam (Figures 3 and 4). The genes *MaoB1* and *Cidec* are known to be negatively regulated by estrogen [53], [54]. Interestingly, downregulation of these genes in Tam + E2-treated mammary in the same manner as in E2-treated mammary suggest that estrogen remains an important regulator of these genes even after co-treatment with Tam (Figure 9). Monoamine oxidase B1 is a ubiquitous enzyme that oxidizes amines including histamine and xenobiotics to prevent chemical toxicity [54]. High concentrations of histamine and thus more proliferation in ductal breast cancer have previously been shown [55]. The increase of histamine concentration in breast cancer cases could be the outcome of either more biosynthesis and/or less metabolism of histamine. Thus, E2-mediated inhibition of *MaoB1* in the mammary of Tam + E2-treated rats (Figure 9) provides advantage to proliferative activities in breast as seen in figures 1E and 5. Cidec is a member of a novel family of cell death-inducing DNA fragmentation factor (DFF) 45-like effectors [CIDEs] and is involved in induction of apoptosis [56], [57]. Therefore, inhibition of Cidec and thus apoptosis in the mammary of Tam + E2-treated rats may help in development of breast cancer (Figures 1F and 9). These data provide support for the idea that endocrine-targeted therapies can lead to activation of novel signaling pathways that circumvent the effects of antiestrogens like Tam. Moreover, estrogen-metabolism mediated oxidative stress can also augment the nongenomic ER actions like activation of downstream signaling kinases such as mitogen-activated protein kinase (MAPK) and AKT, which orchestrate cell proliferation and survival [58], [59]. In addition, these signaling kinases can, in turn, activate ER itself or its coactivator proteins, which augment ER-mediated signaling and promote Tam resistance that ensures the survival of a breast cancer cell even in the presence of Tam [60]. This nongenomic ER action has previously been described in many target organs and tissues including breast [61], [62], [63].

Overall, our results demonstrate that under conditions of long-term chronic E2 exposure, Tam does not provide complete protection against E2-metabolism mediated ROS generation and DNA damage. Our results are in agreement with the role of oxidant stress in estrogen-induced carcinogenesis. Although Tam treatment inhibits estrogen-induced breast tumor development and increases the latency of tumor development, it does not completely abrogate estrogen-dependent breast tumor development in a rat model of estrogen-induced breast carcinogenesis. Thus, lack of complete inhibition of E2-induced breast cancer by Tam may be because of persistent estrogen-induced oxidant stress.

## Materials and Methods

### Ethics statement

The animals were treated and handled according to the guidelines of the Columbia University's Animal Care and Use Committee. The study was approved by the Institutional Animal Care and Use Committee of Columbia University, New York (approval number AC- AAAA9396).

### Treatment of Animals and Histopathological Analysis

Female ACI rats (4 weeks of age; Harlan Sprague Dawley, Indianapolis, IN) were housed under controlled temperature, humidity, and lighting conditions. After a one-week acclimatization period, rats were divided into four groups; i) Control, ii) E2, iii) Tam, and iv) Tam + E2. Rats were implanted subcutaneously with 3 mg E2 and 40 mg Tam as pellets [7], [26]. E2 pellets were prepared in 17 mg cholesterol as a binder as described previously [1], [9], [64]. Control and Tam groups received 17 mg cholesterol pellet. Animals were fed phytoestrogen-free AIN76A diet (Dyets, Bethlehem, PA) and water was given *ad libitum* to all the animals. E2, cholesterol and Tam pellets were prepared using a pellet press as described previously [65]. Rats were treated with Tam or E2 for 240 days (8 months). Animals were palpated twice weekly to monitor mammary tumor development. At the end of 240 days of treatment, animals were anesthetized using isoflurane and euthanized. Mammary, mammary tumors, ovary, kidney, uterus, lung and liver tissues were removed and snap-frozen in liquid nitrogen for further studies. Frozen tissues were stored at −70°C. A portion of all the excised tissues was stored in 10% buffered formalin for histopathologic analyses. The formalin-fixed tissue was embedded in paraffin, and sections of 4 to 5 µm thickness were cut. Paraffin-embedded sections of the mammary, liver, uterus, kidney, lung and ovary were stained with hematoxylin and eosin for histopathologic evaluations. Tumor incidence and the number of tumor nodules per rat were counted at the time of dissection.

### Immunohistochemical Analysis

Paraffin-embedded blocks were sectioned and mounted on frost-free slides. Tissue sections of 4–5 µm thickness were deparaffinized in xylene and rehydrated through a series of graded alcohols. Tissue sections were washed with 1× PBS and endogenous peroxidases were blocked with 0.3% hydrogen peroxide in methanol for 30 min at 25°C. After three 5 min washes in 1× PBS, the sections were incubated for 10 min at 80°C in 10 mmol/L sodium citrate buffer (pH 6.0) for antigen retrieval. Tissue sections were then incubated in blocking solution according to Vectastain Elite ABC kit (Vector laboratories, Burlingame, CA) for 20 min at 25°C in a humidified chamber. Control (no primary antibody) and experimental slides were incubated overnight at 4°C in a humidified chamber in blocking solution with PCNA (1∶200, sc-11407; Santa Cruz Biotechnology, Santa Cruz, CA) or ER-α antibody (1∶200, sc-17774; Santa Cruz Biotechnology, Santa Cruz, CA). After 5 min wash with 1× PBS, biotinylated secondary antibody (Vector laboratories) was added and sections were incubated at 25°C for 2 h and then washed three times with 1× PBS. Tissue sections were then incubated for 30 min with Vectastain Elite ABC reagent (Vector laboratories). After 1× PBS washing, staining was visualized with DAB substrate kit (Vector laboratories) according to supplier's instructions. All sections were counterstained with Harris hematoxylin, dehydrated in ethanol, cleared in xylene and mounted with Permount (Fisher Scientific, Hanover Park, IL). Tissue sections from at least 3 different animals in each group were used for immunostaining. Images were obtained at 100× using a Leica DMI 3000B microscope (Leica Microsystems Inc., Bannockburn, IL).

### Analysis of 8-iso-Prostane F_2α_ (8-isoPGF_2α_) Levels

Total 8-isoPGF_2α_ levels in the mammary and liver tissues of female ACI rats were quantified using a direct 8-isoPGF_2α_ enzyme immunoassay kit (Assay Designs, Ann Arbor, MI), according to the suppliers instructions, as described previously [1], [7, {Singh, 2010 #1122}]. Briefly, rat mammary and liver tissues (50–100 mg) were homogenized in cold PBS (pH 7.4) containing 0.005% butylated hydroxytolulene. Homogenization was carried out in the TissueLyser (Qiagen, Valencia, CA) at 29 cycles per second for 4 min at 1 min intervals. Protein concentrations from the homogenates were determined using the Pierce BCA protein assay kit (Pierce, Rockford, IL). For determination of 8-isoPGF_2α_ levels, 100 µl of the homogenate from each tissue was hydrolyzed by incubation with 25 µl of 10 N NaOH at 45°C for 2 h. The reaction mixture was cooled on ice for 5 min, neutralized with 25 µl of 12 N HCl and centrifuged for 5 min at 4°C. Fifty microliter of the hydrolyzed/neutralized supernatant sample was then used in the 96-well format 8-isoPGF_2α_ assay. Samples were incubated with the 8-isoPGF_2α_ antibody for 18 h at 4°C. The contents of the wells were emptied after incubation and washed with wash buffer. After complete removal of wash buffer from the wells, the color was developed by incubation with 200 µl of p-nitrophenyl phosphate for 45 min, at room temperature. The reaction was stopped by the addition of 50 µl of stop solution and the plate was read at 405 nm. Standards were run on each plate and standard curve was generated by measuring the optical density of 160–100,000 pg/ml of 8-isoPGF_2α_ standards that were processed simultaneously with the unknown samples. Data were expressed as mean 8-isoPGF_2α_ pg/µg protein ± SEM. Fold changes were calculated by comparing 8-isoPGF_2α_ levels detected in the mammary or liver tissue of treated animals to levels in the respective tissue of age-matched control animals.

### Analysis of SOD, GPx and CAT Activity

Total SOD, GPx and CAT activities were quantified using commercially available kits from Cayman Chemical Company (Ann Arbor, MI) and as reported recently [7], [9]. Briefly, the total SOD activity was quantified using a tetrazolium salt for detection of superoxide radicals, generated by xanthine oxidase and hypoxanthine. Approximately 50 mg of mammary or liver tissue was homogenized in 50 mM phosphate buffer containing 1 mM EDTA, 210 mM mannitol and 70 mM sucrose. After homogenization, homogenates were centrifuged at 1500×g for 5 min at 4°C. The supernatant was removed and the protein concentration was measured using the BCA Protein Assay kit (Pierce, Rockland, IL). SOD2 activity was also quantified simultaneously with the total SOD activity using 3 mM potassium cyanide in the assay that inhibits both SOD1 and SOD3 [66]. Standards were run on each plate along with the unknown samples for generation of standard curve. The absorbance of the sample and standards were measured at 450 nm, using a plate reader (Versamax Microplate Reader, Molecular Devices, Sunnyvale, CA). SOD3 activity was quantified using an in-gel activity staining assay as described previously (24). The same protein samples that were used in total SOD activity assay were used for SOD3 in-gel activity assay. The protein samples were separated by polyacrylamide gel electrophoresis. The gel was then soaked in a solution containing nitroblue tetrazolium, riboflavin, and N,N,N′,N′-tetramethylethylenediamine. The gel was then illuminated at 560 nm for 15 min. During light exposure, the area where SOD3 activity was located showed an achromatic band on the gel. After 15 min, the gel image was captured with Alpha Innotech FluorChem HD2 (Alpha Innotech, San Leandro, CA) gel documentation system and quantified using AlphaEase FC StandAlone software (version 6.0.0.14; Alpha Innotech). The activity of SOD3 was determined based on the image intensities produced by the standard of bovine erythrocyte SOD3 (Sigma, St. Louis, MO) in the activity gels. SOD1 activity was obtained by subtracting SOD2 and SOD3 activities from total SOD activity of that particular sample. SOD activities were reported as units/mg protein.

The GPx assay kit measures GPx activity by way of a coupled reaction with glutathione reductase. This assay is based on the production of oxidized glutathione during reduction of hydroperoxide by GPx and then recycling to its reduced state by glutathione reductase and NADPH. The oxidation of NADPH to NADP^+^ causes a decrease in the absorbance at 340 nm. The rate of decrease in the absorbance of the samples at 340 nm is directly proportional to the GPx activity in the sample. At the time of analysis, approximately 50 mg of mammary or liver tissue was homogenized in 50 mM phosphate buffer containing 1 mM EDTA, 210 mM mannitol and 70 mM sucrose. After homogenization, homogenates were centrifuged at 10,000×g for 15 min at 4°C. Seven time points with a gap of 1 min were obtained in order to accurately assess the decrease in absorbance at 340 nm. GPx activity was reported as nmol/min/mg protein.

The determination of CAT activity is based on the reaction of the CAT enzyme with methanol, in the presence of an optimal concentration of H_2_O_2_. The formaldehyde produced is measured spectrophotometrically at 540 nm using 4-amino-3-hydrazino-5-mercapto-1, 2, 4-triazole (Purpald) as the chromogen. Purpald specifically forms a bicyclic heterocycle with aldehydes, which changes from colorless to purple following oxidation. At the time of analysis, approximately 50 mg of mammary or liver tissue was homogenized in 50 mM phosphate buffer containing 1 mM EDTA, 210 mM mannitol and 70 mM sucrose. After homogenization, homogenates were centrifuged at 10,000×g for 15 min at 4°C. Standards were run simultaneously on each plate. CAT activity was reported as nmol/min/mg protein. Fold changes in SOD, GPx and CAT activity were calculated by comparing enzyme activity in the mammary or liver tissue of treated groups to the activity measured in the respective tissue of age-matched respective controls.

### Reverse Transcription and Real-time PCR

Total RNA was isolated from ACI rat mammary and mammary tumors from 8 month treatment groups using RNeasy lipid tissue kit (Qiagen, Valencia, CA) and QIAcube automated machine according to the supplier's protocols. Five microgram total RNA was reverse transcribed using the superscript II reverse transcription system and an oligo-dT_16–18_ primer (Invitrogen, Carlsbad, CA). Real-time PCR was performed using rat specific *PR*, *monoamine oxygenase B1* (*MaoB1*) and *cell death inducing DFF45 like effector C* (*Cidec*) QuantiTect primers and QuantiFast SYBR green master mix (Qiagen, Valencia, CA) in duplicate 25 µl reactions by using iCycler iQ5 system (BioRad Laboratories, Hercules, CA). Data were analyzed from at least 5 different animals from each group using BioRad iQ5 optical system software version 2.0. The expression of cyclophilin, a housekeeping gene, was used for quantification and standardization purposes whose expression has previously been shown not to be altered by estrogen treatment [67]. The expression of gene of interest relative to cyclophilin was determined by dividing the number of cDNA molecules for the gene of interest by the number of cyclophilin cDNA molecules. Standards were created for each gene and a standard curve was run on each plate to allow for accurate quantification of cDNA, as reported previously [67].

### Western Blot Analysis

Approximately 50 µg of ACI rat mammary tumor and mammary tissues were homogenized in a tissue protein extraction buffer (T-PER, Thermo Scientific, IL) using a PRO 200 rotor stator homogenizer (PRO Scientific, CT). After homogenization, samples were centrifuged at 10,000×g and the clear supernatant was saved. The Pierce BCA Protein Assay kit was used to determine the protein concentration of each sample (Pierce, Rockford, IL). Sixty micrograms total protein was size fractionated on a 12% SDS-polyacrylamide gel, and transferred onto a PVDF membrane under standard conditions. Membranes were blocked in 5% dry non-fat milk/PBS/0.05% Tween-20 for 2 h. Cytochrome P450 3A4 (CYP3A4) (BD Biosciences, San Jose, CA), CYP2D6, FMO1, SOD1, SOD2, GPx-1/2 and CAT primary antibodies (Santa Cruz Biotechnology, Santa Cruz, CA) were diluted in PBS/0.05% Tween-20 and used individually for immunodetection. After overnight incubation at room temperature with the primary antibody, membranes were washed four times with 8 min per wash using PBS/0.05% Tween-20. Membranes were then incubated with horse radish peroxidase-conjugated respective secondary antibodies (Santa Cruz Biotechnology, Santa Cruz, CA) diluted in PBS/0.05% Tween-20. After incubation for 2 h at room temperature, the membranes were washed again as described above. Chemiluminescent detection was performed using the BM Chemiluminescence Detection kit (Roche, Indianapolis, IN) and Alpha Innotech FluorChem HD2 (Alpha Innotech, San Leandro, CA) gel documentation system. Membranes probed for desired protein were stripped and washed in PBS/0.05% Tween-20 and reprobed with α-tubulin rat monoclonal antibody (Santa Cruz Biotechnology, Santa Cruz, CA) using the method described above. Protein samples from at least 3 different animals in each group were used for immunoblotting. Intensities of the bands were quantified and normalized using AlphaEase FC StandAlone software (version 6.0.0.14; Alpha Innotech).

### 8-hydroxydeoxyguanosine (8-OHdG) Analysis

8-hydroxydeoxyguanosine, a ubiquitous marker of oxidative DNA damage was estimated in mammary tumors and in mammary tissues of rats treated with E2 in the presence or absence of Tam as well as in age-matched controls. DNA was isolated using DNeasy blood & tissue kit (Qiagen, Valencia, CA) according to the supplier's protocols with some modifications. Diethylenetriamine pentaacetic acid (0.1 mM) and ascorbic acid (2 mM) were used throughout the DNA isolation process to avoid possible spurious DNA oxidation. DNase-free RNase was used to degrade RNA as per supplier's protocol. The RNA-free DNA thus obtained was used to estimate 8-OHdG levels using Oxiselect oxidative DNA damage ELISA kit (Cell Biolabs, San Diego, CA) according to supplier's protocols with some modifications as described earlier [68]. Briefly, 20 µg DNA was digested with 10 U DNase 1 (Qiagen, Valencia, CA) at 37°C for 30 min in presence of 100 mM MgCl_2_ and 1 M Tris-HCl (pH 7.4). After DNA digestion with DNase 1, pH was adjusted to 5.2 with 3 M sodium acetate (pH 5.2) and DNA reaction mixture was subjected to 1 µl of nuclease P1 (1 U/µl) digestion for 1.5 h at 37°C. After 1.5 h incubation, 1 M Tris-HCl (pH 8.0) was used to bring the pH back to 7.4 followed by 1 µl of alkaline phosphatase (1 U/µl stock) treatment for 1 h. The reaction mixture was then subjected to 1 µl of each phosphodiesterase I (0.01 U/µl) and phosphodiesterase II (0.01 U/µl) digestion for 30 min at 37°C. The reaction mixture was centrifuged for 5 min at 6000×g and the supernatant was used for 8-OHdG ELISA assay.

### Statistical Analyses

Statistical analysis was performed by using Sigma Plot 11.0 (Systat Software, San Jose, CA). Fisher's exact test was used to compare tumor incidence between two treatment groups, or between a treatment group and a specific control group. 8-iso-Prostane F_2α_, 8-OHdG, SOD, CAT and GPx assays were all done using at least 8 samples from 8 different animals in each treatment group. The unpaired t-test analysis was used to calculate p values for comparisons between the treated animals and their respective age-matched controls. A p value <0.05 was considered significant.

## References

[1] Bhat HK, Calaf G, Hei TK, Loya T, Vadgama JV (2003). Critical role of oxidative stress in estrogen-induced carcinogenesis.. Proc Natl Acad Sci U S A.

[2] Cavalieri E, Chakravarti D, Guttenplan J, Hart E, Ingle J (2006). Catechol estrogen quinones as initiators of breast and other human cancers: implications for biomarkers of susceptibility and cancer prevention.. Biochim Biophys Acta.

[3] IARC (1999). IARC monographs on the evaluation of carcinogenic risks to humans: hormonal contraception and postmenopausal hormone therapy.. International Agency for Research on Cancer, Lyon.

[4] Cavalieri E, Rogan E (2006). Catechol quinones of estrogens in the initiation of breast, prostate, and other human cancers: keynote lecture.. Ann N Y Acad Sci.

[5] Clemons M, Goss P (2001). Estrogen and the risk of breast cancer.. N Engl J Med.

[6] Liehr JG, Roy D (1990). Free radical generation by redox cycling of estrogens.. Free Radic Biol Med.

[7] Mense SM, Remotti F, Bhan A, Singh B, El-Tamer M (2008). Estrogen-induced breast cancer: alterations in breast morphology and oxidative stress as a function of estrogen exposure.. Toxicol Appl Pharmacol.

[8] Mense SM, Singh B, Remotti F, Liu X, Bhat HK (2009). Vitamin C and alpha-naphthoflavone prevent estrogen-induced mammary tumors and decrease oxidative stress in female ACI rats.. Carcinogenesis.

[9] Singh B, Mense SM, Remotti F, Liu X, Bhat HK (2009). Antioxidant butylated hydroxyanisole inhibits estrogen-induced breast carcinogenesis in female ACI rats.. J Biochem Mol Toxicol.

[10] Fisher B, Costantino JP, Wickerham DL, Redmond CK, Kavanah M (1998). Tamoxifen for prevention of breast cancer: report of the National Surgical Adjuvant Breast and Bowel Project P-1 Study.. J Natl Cancer Inst.

[11] Wilson AJ, Baum M, Brinkley DM, Dossett JA, McPherson K (1985). Six-year results of a controlled trial of tamoxifen as single adjuvant agent in management of early breast cancer.. World J Surg.

[12] Brzozowski AM, Pike AC, Dauter Z, Hubbard RE, Bonn T (1997). Molecular basis of agonism and antagonism in the oestrogen receptor.. Nature.

[13] Group EBCTC (1998). Tamoxifen for early breast cancer: an overview of the randomised trials.. Lancet.

[14] Riggins RB, Lan JP, Zhu Y, Klimach U, Zwart A (2008). ERRgamma mediates tamoxifen resistance in novel models of invasive lobular breast cancer.. Cancer Res.

[15] Carthew P, Martin EA, White IN, De Matteis F, Edwards RE (1995). Tamoxifen induces short-term cumulative DNA damage and liver tumors in rats: promotion by phenobarbital.. Cancer Res.

[16] Seoud MA, Johnson J, Weed JC (1993). Gynecologic tumors in tamoxifen-treated women with breast cancer.. Obstet Gynecol.

[17] Clarke R, Liu MC, Bouker KB, Gu Z, Lee RY (2003). Antiestrogen resistance in breast cancer and the role of estrogen receptor signaling.. Oncogene.

[18] Riggins RB, Bouton AH, Liu MC, Clarke R (2005). Antiestrogens, aromatase inhibitors, and apoptosis in breast cancer.. Vitam Horm.

[19] Riggins RB, Schrecengost RS, Guerrero MS, Bouton AH (2007). Pathways to tamoxifen resistance.. Cancer Lett.

[20] Phillips DH, Carmichael PL, Hewer A, Cole KJ, Poon GK (1994). alpha-Hydroxytamoxifen, a metabolite of tamoxifen with exceptionally high DNA-binding activity in rat hepatocytes.. Cancer Res.

[21] Styles JA, Davies A, Lim CK, De Matteis F, Stanley LA (1994). Genotoxicity of tamoxifen, tamoxifen epoxide and toremifene in human lymphoblastoid cells containing human cytochrome P450s.. Carcinogenesis.

[22] Lim CK, Yuan ZX, Lamb JH, White IN, De Matteis F (1994). A comparative study of tamoxifen metabolism in female rat, mouse and human liver microsomes.. Carcinogenesis.

[23] Crespi CL, Gonzalez FJ, Steimel DT, Turner TR, Gelboin HV (1991). A metabolically competent human cell line expressing five cDNAs encoding procarcinogen-activating enzymes: application to mutagenicity testing.. Chem Res Toxicol.

[24] Han XL, Liehr JG (1992). Induction of covalent DNA adducts in rodents by tamoxifen.. Cancer Res.

[25] Umemoto A, Monden Y, Suwa M, Kanno Y, Suzuki M (2000). Identification of hepatic tamoxifen-DNA adducts in mice: alpha-(N(2)-deoxyguanosinyl)tamoxifen and alpha-(N(2)-deoxyguanosinyl)tamoxifen N-oxide.. Carcinogenesis.

[26] Li SA, Weroha SJ, Tawfik O, Li JJ (2002). Prevention of solely estrogen-induced mammary tumors in female aci rats by tamoxifen: evidence for estrogen receptor mediation.. J Endocrinol.

[27] Arnerlov C, Emdin SO, Cajander S, Bengtsson NO, Tavelin B (2001). Intratumoral variations in DNA ploidy and s-phase fraction in human breast cancer.. Anal Cell Pathol.

[28] Li JJ, Papa D, Davis MF, Weroha SJ, Aldaz CM (2002). Ploidy differences between hormone- and chemical carcinogen-induced rat mammary neoplasms: comparison to invasive human ductal breast cancer.. Mol Carcinog.

[29] Harvell DM, Strecker TE, Tochacek M, Xie B, Pennington KL (2000). Rat strain-specific actions of 17beta-estradiol in the mammary gland: correlation between estrogen-induced lobuloalveolar hyperplasia and susceptibility to estrogen-induced mammary cancers.. Proc Natl Acad Sci U S A.

[30] Shull JD, Spady TJ, Snyder MC, Johansson SL, Pennington KL (1997). Ovary-intact, but not ovariectomized female ACI rats treated with 17beta-estradiol rapidly develop mammary carcinoma.. Carcinogenesis.

[31] Singh B, Mense SM, Bhat NK, Putty S, Guthiel WA (2010). Dietary quercetin exacerbates the development of estrogen-induced breast tumors in female ACI rats.. Toxicol Appl Pharmacol.

[32] Li JJ, Weroha SJ, Lingle WL, Papa D, Salisbury JL (2004). Estrogen mediates Aurora-A overexpression, centrosome amplification, chromosomal instability, and breast cancer in female ACI rats.. Proc Natl Acad Sci U S A.

[33] Makris A, Allred DC, Powles TJ, Dowsett M, Fernando IN (1997). Cytological evaluation of biological prognostic markers from primary breast carcinomas.. Breast Cancer Res Treat.

[34] Weroha SJ, Li SA, Tawfik O, Li JJ (2006). Overexpression of cyclins D1 and D3 during estrogen-induced breast oncogenesis in female ACI rats.. Carcinogenesis.

[35] Kariagina A, Xie J, Leipprandt JR, Haslam SZ (2010). Amphiregulin Mediates Estrogen, Progesterone, and EGFR Signaling in the Normal Rat Mammary Gland and in Hormone-Dependent Rat Mammary Cancers.. Horm Cancer.

[36] Mukherjee S, Conrad SE (2005). c-Myc suppresses p21WAF1/CIP1 expression during estrogen signaling and antiestrogen resistance in human breast cancer cells.. J Biol Chem.

[37] Patel MM, Bhat HK (2004). Differential oxidant potential of carcinogenic and weakly carcinogenic estrogens: Involvement of metabolic activation and cytochrome P450.. J Biochem Mol Toxicol.

[38] Cavalieri EL, Rogan EG (2004). A unifying mechanism in the initiation of cancer and other diseases by catechol quinones.. Ann N Y Acad Sci.

[39] Li JJ, Li SA (1987). Estrogen carcinogenesis in Syrian hamster tissues: role of metabolism.. Fed Proc.

[40] Liehr JG, Fang WF, Sirbasku DA, Ari-Ulubelen A (1986). Carcinogenicity of catechol estrogens in Syrian hamsters.. J Steroid Biochem.

[41] Yager JD (2000). Endogenous estrogens as carcinogens through metabolic activation.. J Natl Cancer Inst Monogr.

[42] Vitseva O, Flockhart DA, Jin Y, Varghese S, Freedman JE (2005). The effects of tamoxifen and its metabolites on platelet function and release of reactive oxygen intermediates.. J Pharmacol Exp Ther.

[43] Mobley JA, Brueggemeier RW (2004). Estrogen receptor-mediated regulation of oxidative stress and DNA damage in breast cancer.. Carcinogenesis.

[44] Montano MM, Chaplin LJ, Deng H, Mesia-Vela S, Gaikwad N (2007). Protective roles of quinone reductase and tamoxifen against estrogen-induced mammary tumorigenesis.. Oncogene.

[45] Boocock DJ, Brown K, Gibbs AH, Sanchez E, Turteltaub KW (2002). Identification of human CYP forms involved in the activation of tamoxifen and irreversible binding to DNA.. Carcinogenesis.

[46] Dehal SS, Kupfer D (1997). CYP2D6 catalyzes tamoxifen 4-hydroxylation in human liver.. Cancer Res.

[47] Desta Z, Ward BA, Soukhova NV, Flockhart DA (2004). Comprehensive evaluation of tamoxifen sequential biotransformation by the human cytochrome P450 system in vitro: prominent roles for CYP3A and CYP2D6.. J Pharmacol Exp Ther.

[48] Hu Y, Dehal SS, Hynd G, Jones GB, Kupfer D (2003). CYP2D6-mediated catalysis of tamoxifen aromatic hydroxylation with an NIH shift: similar hydroxylation mechanism in chicken, rat and human liver microsomes.. Xenobiotica.

[49] Krueger SK, Vandyke JE, Williams DE, Hines RN (2006). The role of flavin-containing monooxygenase (FMO) in the metabolism of tamoxifen and other tertiary amines.. Drug Metab Rev.

[50] Parte P, Kupfer D (2005). Oxidation of tamoxifen by human flavin-containing monooxygenase (FMO) 1 and FMO3 to tamoxifen-N-oxide and its novel reduction back to tamoxifen by human cytochromes P450 and hemoglobin.. Drug Metab Dispos.

[51] Lewis JS, Jordan VC (2005). Selective estrogen receptor modulators (SERMs): mechanisms of anticarcinogenesis and drug resistance.. Mutat Res.

[52] Shiau AK, Barstad D, Loria PM, Cheng L, Kushner PJ (1998). The structural basis of estrogen receptor/coactivator recognition and the antagonism of this interaction by tamoxifen.. Cell.

[53] Lundholm L, Putnik M, Otsuki M, Andersson S, Ohlsson C (2008). Effects of estrogen on gene expression profiles in mouse hypothalamus and white adipose tissue: target genes include glutathione peroxidase 3 and cell death-inducing DNA fragmentation factor, alpha-subunit-like effector A.. J Endocrinol.

[54] Zhang Z, Chen K, Shih JC, Teng CT (2006). Estrogen-related receptors-stimulated monoamine oxidase B promoter activity is down-regulated by estrogen receptors.. Mol Endocrinol.

[55] von Mach-Szczypinski J, Stanosz S, Sieja K, Stanosz M (2009). Metabolism of histamine in tissues of primary ductal breast cancer.. Metabolism.

[56] Inohara N, Koseki T, Chen S, Wu X, Nunez G (1998). CIDE, a novel family of cell death activators with homology to the 45 kDa subunit of the DNA fragmentation factor.. EMBO J.

[57] Liang L, Zhao M, Xu Z, Yokoyama KK, Li T (2003). Molecular cloning and characterization of CIDE-3, a novel member of the cell-death-inducing DNA-fragmentation-factor (DFF45)-like effector family.. Biochem J.

[58] Clark AS, West K, Streicher S, Dennis PA (2002). Constitutive and inducible Akt activity promotes resistance to chemotherapy, trastuzumab, or tamoxifen in breast cancer cells.. Mol Cancer Ther.

[59] Lee YJ, Galoforo SS, Berns CM, Chen JC, Davis BH (1998). Glucose deprivation-induced cytotoxicity and alterations in mitogen-activated protein kinase activation are mediated by oxidative stress in multidrug-resistant human breast carcinoma cells.. J Biol Chem.

[60] Shou J, Massarweh S, Osborne CK, Wakeling AE, Ali S (2004). Mechanisms of tamoxifen resistance: increased estrogen receptor-HER2/neu cross-talk in ER/HER2-positive breast cancer.. J Natl Cancer Inst.

[61] Dhandapani KM, Brann DW (2002). Protective effects of estrogen and selective estrogen receptor modulators in the brain.. Biol Reprod.

[62] Kousteni S, Bellido T, Plotkin LI, O'Brien CA, Bodenner DL (2001). Nongenotropic, sex-nonspecific signaling through the estrogen or androgen receptors: dissociation from transcriptional activity.. Cell.

[63] Pedram A, Razandi M, Aitkenhead M, Hughes CC, Levin ER (2002). Integration of the non-genomic and genomic actions of estrogen. Membrane-initiated signaling by steroid to transcription and cell biology.. J Biol Chem.

[64] Han X, Liehr JG (1994). DNA single-strand breaks in kidneys of Syrian hamsters treated with steroidal estrogens: hormone-induced free radical damage preceding renal malignancy.. Carcinogenesis.

[65] Bhat HK, Hacker HJ, Bannasch P, Thompson EA, Liehr JG (1993). Localization of estrogen receptors in interstitial cells of hamster kidney and in estradiol-induced renal tumors as evidence of the mesenchymal origin of this neoplasm.. Cancer Res.

[66] Marklund S (1980). Distribution of CuZn superoxide dismutase and Mn superoxide dismutase in human tissues and extracellular fluids.. Acta Physiol Scand.

[67] Bhat HK, Epelboym I (2004). Suppression of calbindin D28K in estrogen-induced hamster renal tumors.. J Steroid Biochem Mol Biol.

[68] Huang X, Powell J, Mooney LA, Li C, Frenkel K (2001). Importance of complete DNA digestion in minimizing variability of 8-oxo-dG analyses.. Free Radic Biol Med.

